# Maine

**Published:** 1899-08

**Authors:** I. L. Salley

**Affiliations:** Secretary


### MAINE VETERINARY MEDICAL ASSOCIATION.

THE regular meeting of this Association was held at Bangor July 12th. In the afternoon a clinic was held at the office of Dr. Murch. Numerous operations were performed. Dr. Murch demonstrated a very nice method for ovariotomy in the bitch, besides some minor operations, as castration of cryptorchids, and tenotomy, were performed.

At 7.30 P.M. a meeting was held at the Bangor House; Dr. Russell was elected chairman *pro. tem.* Drs. Cleaver, Russell, Joly, Salley, Freeman, Murch, and Dwinal responded to roll-call. The minutes of the previous meeting were read and accepted. The financial condition of the society was discussed and the Secretary instructed to collect all dues. Voted to hold a special meeting at New York, September 5th. Dr. Joly was elected a representative to the meeting at New York of the American Veterinary Medical Association in September. Moved to adjourn to meet at Waterville in October.

I. L. SALLEY, D.V.S.,
Secretary.

